# Response of phytohormone mediated plant homeodomain (PHD) family to abiotic stress in upland cotton (*Gossypium hirsutum* spp.)

**DOI:** 10.1186/s12870-020-02787-5

**Published:** 2021-01-06

**Authors:** Huanhuan Wu, Lei Zheng, Ghulam Qanmber, Mengzhen Guo, Zhi Wang, Zuoren Yang

**Affiliations:** 1 State Key Laboratory of Cotton Biology, Cotton Research Institute of Chinese Academy of Agricultural Sciences, Anyang, 455000 Henan China; 2 National Key Laboratory of Crop Genetic Improvement, Huazhong Agricultural University, Wuhan, 430070 Hubei China; 3 Zhengzhou Research Base, State Key Laboratory of Cotton Biology, Zhengzhou University, Zhengzhou, 450001 Henan China

**Keywords:** Cotton, PHD, Transcription factor, Phytohormone, Stress tolerance, Co-expression network, Transcriptome analysis

## Abstract

**Background:**

The sequencing and annotations of cotton genomes provide powerful theoretical support to unravel more physiological and functional information. Plant homeodomain (PHD) protein family has been reported to be involved in regulating various biological processes in plants. However, their functional studies have not yet been carried out in cotton.

**Results:**

In this study, 108, 55, and 52 *PHD* genes were identified in *G. hirsutum*, *G. raimondii*, and *G. arboreum*, respectively. A total of 297 *PHD* genes from three cotton species, *Arabidopsis*, and rice were divided into five groups. We performed chromosomal location, phylogenetic relationship, gene structure, and conserved domain analysis for *GhPHD* genes. *GhPHD* genes were unevenly distributed on each chromosome. However, more *GhPHD* genes were distributed on At_05, Dt_05, and At_07 chromosomes. GhPHD proteins depicted conserved domains, and *GhPHD* genes exhibiting similar gene structure were clustered together. Further, whole genome duplication (WGD) analysis indicated that purification selection greatly contributed to the functional maintenance of *GhPHD* gene family. Expression pattern analysis based on RNA-seq data showed that most *GhPHD* genes showed clear tissue-specific spatiotemporal expression patterns elucidating the multiple functions of *GhPHDs* in plant growth and development. Moreover, analysis of *cis*-acting elements revealed that *GhPHDs* may respond to a variety of abiotic and phytohormonal stresses. In this regard, some *GhPHD* genes showed good response against abiotic and phytohormonal stresses. Additionally, co-expression network analysis indicated that *GhPHDs* are essential for plant growth and development, while *GhPHD* genes response against abiotic and phytohormonal stresses may help to improve plant tolerance in adverse environmental conditions.

**Conclusion:**

This study will provide useful information to facilitate further research related to the vital roles of *GhPHD* gene family in plant growth and development.

## Background

Plants often face various abiotic and biotic stress conditions. Abiotic stresses include heat, cold, drought, and salinity, whereas biotic stresses mainly come from bacteria, fungi, viruses, and insects. These abiotic and biotic stresses significantly reduce crop quality and productivity world-wide [1, 2]. In order to adapt such unfavorable environment, plants have established a comprehensive mechanism to combat stress signals and mitigate their effects on plant growth and development [3]. Phytohormones play significant roles in regulating developmental processes and signal transduction networks, which respond to various abiotic stresses. Brassinosteroid (BR), jasmonate (JA), gibberellin (GA), salicylic acid (SA), auxin, and abscisic acid (ABA) regulate plant growth, development, stress, and defense responses [4–11], but how phytohormones mediate the growth and stress trade-off is unclear.

Zinc finger protein motifs are part of many protein families and widely distributed in eukaryotic organisms. The term “zinc finger” represents the sequence motif in which cysteines and/or histidines coordinate the zinc atom(s) to form the local peptide structure that are required for their specific functions. The “finger” structural motif has been divided into different types, such as TFIIIA-type zinc finger (EPF1, SUPERMAN) [12, 13], WRKY family (WRKY1, 2, and 3), GATA1-type protein (NTL1) [14, 15], Dof family (Dof1) [16, 17], RING-finger type (COP1) [18], PHD-finger family (AtHAT3.1 and ZmHOX1a) [19, 20], LIM family (SF3) [21, 22], and other uncategorized types. Plant homeodomain (PHD) zinc fingers are small reader domains found in several chromatin-binding proteins. In plants, PHD proteins are usually zinc finger proteins with one or more PHD domains, which have a Cys4-His-Cys3 zinc-binding motif consisting of about 60 amino acids [23]. It is worth noting that the number of amino acids between cysteine and histidine or between cysteine residues in the PHD domain are conserved, while second amino acid (before the penultimate cysteine residue) is usually an aromatic amino acid, such as tryptophan [24].

Since the discovery of the first PHD protein HAT3.1 (Histone acetyltransferase 3.1) in *Arabidopsis*, more PHD proteins have been identified to participate in many physiological and biochemical processes involved in the structure and transcription of chromatin [25]. In *Arabidopsis*, PHD protein MMD1 (Male meiocyte death 1)/DUET is specifically expressed in male meiocytes and involved in regulating gene expression during meiosis, mutations of *mmd1* gene leads to the death of male meiotic cells [26–28]. Epigenetic regulation in eukaryotes is performed through complex signal interactions between chromatin markers and small RNA species. *AtVIM1* (Variant in methylation 1) functions in DNA methylation-histone interface to maintain the centromeric heterochromation in *Arabidopsis* [29]. In addition, PHD proteins are involved in regulating plant response to abiotic stresses and altering plant growth and development [30, 31]. In soybean, six Alfin1-type PHD proteins were identified to respond against salt, cold, drought, and ABA treatment. For instance, *GmPHD2* improve salt tolerance in transgenic *Arabidopsis* plants compared with the wild type plants [32]. In *Arabidopsis*, AtVIN3 (Vernalization insensitive 3) protein binds to modified histone in vitro to change the binding specificity of PHD-finger domain and accelerate the vernalization reaction in vivo [33]. During seed germination, the *AL PHD-PRC1* complex affect seed developmental genes from the active state associated with H3K4me3 to the repressive transcriptional state associated with H3K27me3, thereby promote seed germination [34]. PHD protein GSR1 (Germostatin resistance locus 1) is a member of auxin-mediated genetic network for seed germination and form a corepressor with *ARF16* (Auxin response factor 16) to regulate seed germination [35]. Therefore, PHD proteins play irreplaceable roles in the biological processes of life.

At present, the PHD protein family has been studied in several plants, such as *Arabidopsis thaliana*, poplar (*Populus trichocarpa*) [36], maize (*Zea mays*) [30], moso bamboo (*Phyllostachys edulis*) [37], carrot (*Daucus carota* L.) [38], potato (*Solanum tuberosum*) [39], and pear (*Pyrus bretschneideri*) [40]. However, comprehensive identification and characterization of cotton PHD protein family has not been carried out till date. Upland cotton (*Gossypium hirsutum*) is the most important natural fiber crop in the world. Recently, the availability of the complete genome sequence and annotations of *G. hirsutum* [41], *G. arboreum* [42], and *G. raimondii* [43] provided an excellent opportunity to identify and characterize *PHD* transcription factors in cotton. In this study, we performed the whole genome-wide analysis, tissue expression pattern analysis, relative expression level analysis under different stresses and phytohormones treatment, and co-expression network analysis of *GhPHD* genes in upland cotton. Our results indicated that *GhPHD* genes are involved in various processes of plant growth and development, and phytohormones mediate responses of *GhPHD* genes against abiotic stresses.

## Results

### Genome-wide identification of PHD proteins in cotton

Based on the homology of protein sequences, 108, 52, and 55 PHD proteins were identified in three cotton species *G. hirsutum*, *G. arboreum*, and *G. raimondii*, respectively. In addition, 39 and 43 PHD proteins were identified in *Arabidopsis* and rice, respectively (Table S1). Among 108 GhPHD proteins, 56 members belong to the At subgenome and 52 members belong to the Dt subgenome. The predicted biophysical characteristic of *GhPHDs* (Table 1) indicates that the length of GhPHD proteins ranges from 159 aa (GhPHD28) to 2231 aa (GhPHD39) with an average length of 741 aa. Moreover, the molecular weight of GhPHD proteins ranges from 17.76 kD (GhPHD28) to 247.42 kD (GhPHD39) with an average value of 93.09 kD. The isoelectric point (pI) of GhPHD proteins ranges from 4.58 (GhPHD38) to 10.41 (GhPHD103) with an average value of 6.89. Furthermore, the predicted subcellular localization indicated that 93 GhPHD proteins are located in nucleus, ten in cytoplasm, and five are extracellular.

**Table 1:** Physicochemical parameters of 108 *GhPHD* genes in *G. hirsutum*

Name	Protein length (aa)	Molecular weight (kDa)	Charge	Isoelectric point	Grand average of hydropathy	Subcellular localization
GhPHD1	217	24.915	5	7.895	− 0.694	Nuclear
GhPHD2	1033	114.441	32.5	8.49	−0.274	Nuclear
GhPHD3	1030	114.182	36	8.594	−0.306	Nuclear
GhPHD4	815	90.024	5	6.895	−0.323	Nuclear
GhPHD5	1303	144.878	−9.5	6.002	−0.713	Nuclear
GhPHD6	700	79.363	−4.5	6.21	−0.308	Nuclear
GhPHD7	700	79.363	−4.5	6.21	−0.308	Nuclear
GhPHD8	345	39.474	12	8.648	−0.573	Nuclear
GhPHD9	251	28.253	−8	4.891	−0.621	Nuclear
GhPHD10	786	86.704	−35.5	4.631	−0.966	Nuclear
GhPHD11	216	24.846	8.5	8.262	−0.789	Nuclear
GhPHD12	375	42.862	5.5	7.542	−0.708	Nuclear
GhPHD13	237	26.757	−4	5.421	−0.596	Nuclear
GhPHD14	959	104.731	−3	6.227	−1.126	Nuclear
GhPHD15	733	82.869	40.5	9.936	−0.907	Nuclear
GhPHD16	252	28.482	−8.5	4.84	−0.717	Nuclear
GhPHD17	1680	189.096	46.5	8.1	−0.669	Nuclear
GhPHD18	252	28.35	−7.5	4.894	−0.661	Nuclear
GhPHD19	238	27.277	4.5	7.669	−0.648	Cytoplasmic
GhPHD20	493	55.306	5	7.03	−0.485	Nuclear
GhPHD21	600	67.261	5.5	7.049	−0.611	Nuclear
GhPHD22	1084	122.987	34	8.276	−0.574	Nuclear
GhPHD23	253	28.577	−5.5	5.132	−0.736	Nuclear
GhPHD24	259	29.239	−7.5	4.915	−0.708	Nuclear
GhPHD25	224	25.723	6.5	8.087	−0.682	Cytoplasmic
GhPHD26	870	95.472	3	6.876	−0.472	Nuclear
GhPHD27	1358	154.506	35	7.891	−0.677	Nuclear
GhPHD28	159	17.763	0	6.496	−0.666	Extracellular
GhPHD29	733	80.954	22	8.271	−0.664	Nuclear
GhPHD30	1247	141.67	24	7.655	−0.43	Nuclear
GhPHD31	949	104.907	2.5	6.779	−0.416	Nuclear
GhPHD32	1618	180.35	45	8.404	−0.446	Nuclear
GhPHD33	1618	180.725	41.5	8.289	−0.442	Nuclear
GhPHD34	216	24.95	8.5	8.399	−0.783	Nuclear
GhPHD35	321	35.88	−4.5	5.599	−0.049	Extracellular
GhPHD36	822	88.768	2	6.651	−0.539	Nuclear
GhPHD37	1305	143.316	−33.5	4.951	−0.624	Nuclear
GhPHD38	705	78.949	22	8.309	−0.315	Extracellular
GhPHD39	2231	247.421	−36	5.321	−0.444	Nuclear
GhPHD40	226	25.942	6	8.086	−0.788	Nuclear
GhPHD41	1685	187.671	−4.5	6.321	−0.389	Nuclear
GhPHD42	1239	138.122	−0.5	6.487	−0.735	Nuclear
GhPHD43	253	28.585	−6	5.13	−0.76	Nuclear
GhPHD44	531	58.455	−4	5.77	−0.564	Nuclear
GhPHD45	389	44.625	26.5	9.906	−0.405	Nuclear
GhPHD46	803	90.452	−16.5	5.132	−0.836	Nuclear
GhPHD47	851	94.805	−26	4.895	−1.011	Nuclear
GhPHD48	212	23.802	26	10.41	−0.745	Nuclear
GhPHD49	1019	116.396	18	7.609	−0.584	Nuclear
GhPHD50	655	74.308	11	7.433	−0.207	Cytoplasmic
GhPHD51	1091	124.448	45	8.516	−0.59	Nuclear
GhPHD52	237	26.997	−3.5	5.244	−0.666	Nuclear
GhPHD53	216	24.723	6.5	8.049	−0.785	Nuclear
GhPHD54	716	81.912	−38.5	4.581	−1.1	Nuclear
GhPHD55	1367	152.049	3	6.689	−0.451	Nuclear
GhPHD56	254	28.492	−6.5	5.136	−0.576	Cytoplasmic
GhPHD57	217	24.929	5	7.895	−0.684	Nuclear
GhPHD58	1031	114.246	33.5	8.459	−0.299	Nuclear
GhPHD59	1031	113.878	36.5	8.592	−0.306	Nuclear
GhPHD60	1299	144.45	−11.5	5.886	−0.711	Nuclear
GhPHD61	290	32.821	−8.5	4.832	−0.419	Nuclear
GhPHD62	216	24.835	8.5	8.262	−0.789	Nuclear
GhPHD63	699	79.319	−4.5	6.21	−0.283	Nuclear
GhPHD64	345	39.311	13	8.745	−0.544	Nuclear
GhPHD65	1084	123.101	34	8.275	−0.579	Nuclear
GhPHD66	237	26.615	−2	5.973	−0.561	Nuclear
GhPHD67	945	103.676	−2	6.329	−1.136	Nuclear
GhPHD68	684	77.195	23	9.073	−0.859	Nuclear
GhPHD69	733	83.057	34.5	9.713	−0.91	Nuclear
GhPHD70	252	28.414	−8.5	4.84	−0.697	Nuclear
GhPHD71	1731	194.29	53	8.282	−0.675	Nuclear
GhPHD72	252	28.35	−7.5	4.894	−0.661	Nuclear
GhPHD73	224	25.664	7.5	8.248	−0.774	Cytoplasmic
GhPHD74	367	41.019	5	7.341	−0.49	Nuclear
GhPHD75	601	67.379	2	6.696	−0.611	Nuclear
GhPHD76	241	27.506	−0.5	6.269	−0.502	Extracellular
GhPHD77	253	28.677	−5.5	5.139	−0.752	Nuclear
GhPHD78	252	28.407	−7.5	4.889	−0.682	Nuclear
GhPHD79	186	21.734	1.5	6.851	−0.752	Cytoplasmic
GhPHD80	237	27.077	−4	5.221	−0.689	Extracellular
GhPHD81	1356	154.398	31	7.737	−0.674	Nuclear
GhPHD82	236	26.789	−12	4.605	−0.598	Cytoplasmic
GhPHD83	676	74.819	22.5	8.253	−0.665	Nuclear
GhPHD84	1382	156.796	34	7.925	−0.467	Nuclear
GhPHD85	949	104.967	2.5	6.779	−0.426	Nuclear
GhPHD86	1653	183.656	37.5	8.15	−0.449	Nuclear
GhPHD87	1618	180.589	39	8.234	−0.447	Nuclear
GhPHD88	216	24.836	5.5	7.902	−0.775	Cytoplasmic
GhPHD89	822	88.688	0	6.506	−0.531	Nuclear
GhPHD90	1301	142.837	−35.5	4.873	−0.619	Nuclear
GhPHD91	705	78.888	23.5	8.373	−0.305	Nuclear
GhPHD92	2182	241.654	−34	5.362	−0.441	Nuclear
GhPHD93	226	25.984	6	8.086	−0.767	Nuclear
GhPHD94	1685	187.566	−4	6.345	−0.397	Nuclear
GhPHD95	1237	137.855	−0.5	6.486	−0.718	Nuclear
GhPHD96	253	28.613	−6	5.13	−0.75	Nuclear
GhPHD97	696	78.217	18.5	8.023	−0.17	Nuclear
GhPHD98	503	55.441	−5.5	5.455	−0.597	Nuclear
GhPHD99	385	44.394	31	10.216	−0.428	Nuclear
GhPHD100	812	91.657	−29	4.818	−0.838	Nuclear
GhPHD101	859	95.86	−32.5	4.789	−1.023	Nuclear
GhPHD102	1019	116.3	19	7.67	−0.593	Nuclear
GhPHD103	655	74.268	11.5	7.443	−0.219	Cytoplasmic
GhPHD104	1091	124.625	43	8.46	−0.581	Nuclear
GhPHD105	889	101.493	−32	4.811	−0.882	Nuclear
GhPHD106	1305	145.225	9.5	7.121	−0.424	Nuclear
GhPHD107	801	90.038	−12.5	5.251	−0.805	Nuclear
GhPHD108	252	28.259	−6.5	5.136	−0.619	Cytoplasmic

### Phylogenetic analysis, chromosomal location, and gene duplication

**Fig. 1:**
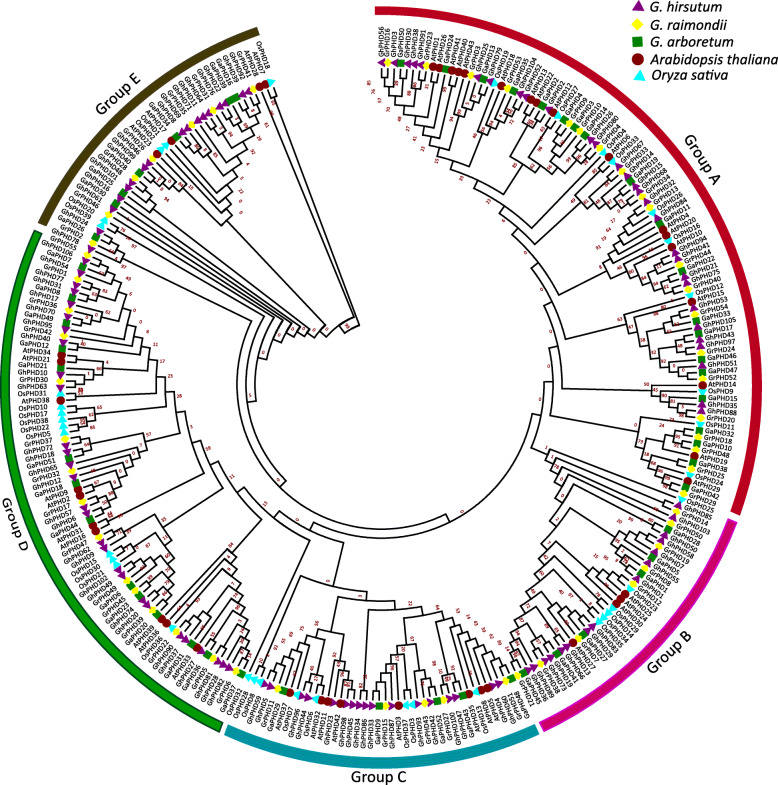
Phylogenetic tree displaying relationships between 108 *G. hirsutum*, 52 *G. arboreum*, 55 *G. raimondii*, 39 *O. sativa* and 43 *A. thaliana* PHD proteins. The phylogenetic tree was constructed in MEGA 6.0 using the neighbor-joining method. The bootstrap test was performed with 1000 iterations. The five subgroups are shown with different colours. At, *Arabidopsis thaliana*; Ga, *Gossypium arboreum*; Gr, *Gossypium raimondii*; Gh, *Gossypium hirsutum*; Os, *Oryza sativa*

In order to understand the phylogenetic relationship of PHD proteins in rice, *Arabidopsis*, and cotton, we constructed a NJ phylogenetic tree and classified PHD proteins into five groups (A-E) (Fig. 1). Among them, most of the orthologous PHD proteins between the diploid and allotetraploid cotton are grouped in same clade exhibiting maximum homology in phylogenetic relationship. Each group contains PHD proteins of these five species, of which group A and D are the first and second largest groups, containing 97 and 79 members, respectively. While, there are relatively few PHD members in groups B, C, and E. Chromosome location analysis showed that 108 *GhPHD* genes are positioned on 26 chromosomes, including 13 chromosomes from the At subgenome and 13 chromosomes from the Dt subgenome (Fig. S1 and Table S2). Deeper insights indicated that At_05, At_07, and Dt_05 chromosomes contain more number of genes (eight *GhPHD* genes on each) and display a dense distribution at the top. However, some chromosomes contain only two *GhPHD* genes, such as At_10, At_11, Dt_03, and Dt_11.

We further investigated the whole genome duplication (WGD) event experienced by *GhPHD* genes. As a result, 73 *GhPHD* gene pairs depict segmental duplication and four gene pairs show tandem duplication events (Table 2), indicating that WGD is the main contributor of *GhPHD* gene family expansion. Duplication gene pairs may have undergone three alternative fates during the evolution process, namely non-functionalization, neo-functionalization, and sub-functionalization [44]. In order to study the evolutionary history of *GhPHD* genes, the *Ka/Ks* calculator 2.0 is used to calculate the synonymous and non-synonymous substitution rates. The *Ka/Ks* ratio of 76 duplicated gene pairs is less than 1, indicating that *GhPHD* genes underwent purification selection pressure with limited functional divergence. However, there is only one gene pair with the *Ka/Ks* greater than 1, indicating the occurrence of positive selection pressure. Collectively, these results indicated that the great contribution of purification selection pressure in the functional maintenance of *GhPHD* genes in upland cotton.

**Table 2:** *Ka/Ks* analysis for the duplicated *PHD* gene pairs from *G. hirsutum*

Duplicated gene 1	Duplicated gene 2	Ka	Ks	Ka/Ks	Purifying selection	Duplicate type
GhPHD1	GhPHD11	0.064	0.533	0.119	Yes	Segmental
GhPHD1	GhPHD62	0.064	0.533	0.119	Yes	Segmental
GhPHD2	GhPHD58	0.011	0.043	0.248	Yes	Segmental
GhPHD5	GhPHD60	0.016	0.039	0.411	Yes	Segmental
GhPHD5	GhPHD95	0.126	0.389	0.324	Yes	Segmental
GhPHD6	GhPHD63	0.004	0.048	0.089	Yes	Segmental
GhPHD9	GhPHD61	0.007	0.032	0.212	Yes	Segmental
GhPHD10	GhPHD14	0.232	0.441	0.526	Yes	Segmental
GhPHD10	GhPHD47	0.245	0.488	0.502	Yes	Segmental
GhPHD10	GhPHD67	0.241	0.471	0.512	Yes	Segmental
GhPHD10	GhPHD101	0.235	0.446	0.527	Yes	Segmental
GhPHD11	GhPHD53	0.039	0.606	0.064	Yes	Segmental
GhPHD11	GhPHD62	0.004	0.014	0.282	Yes	Segmental
GhPHD13	GhPHD66	0.009	0.053	0.169	Yes	Segmental
GhPHD14	GhPHD47	0.376	0.666	0.564	Yes	Segmental
GhPHD14	GhPHD67	0.018	0.046	0.387	Yes	Segmental
GhPHD15	GhPHD68	0.030	0.063	0.468	Yes	Segmental
GhPHD16	GhPHD28	0.089	0.311	0.285	Yes	Segmental
GhPHD16	GhPHD77	0.045	0.352	0.129	Yes	Segmental
GhPHD16	GhPHD82	0.053	0.405	0.130	Yes	Segmental
GhPHD17	GhPHD71	0.008	0.034	0.235	Yes	Segmental
GhPHD17	GhPHD81	0.075	0.381	0.196	Yes	Segmental
GhPHD19	GhPHD25	0.081	0.447	0.180	Yes	Segmental
GhPHD19	GhPHD40	0.083	0.451	0.184	Yes	Segmental
GhPHD19	GhPHD73	0.026	0.053	0.492	Yes	Segmental
GhPHD19	GhPHD79	0.083	0.496	0.167	Yes	Segmental
GhPHD19	GhPHD93	0.085	0.451	0.188	Yes	Segmental
GhPHD20	GhPHD74	0.024	0.032	0.753	Yes	Segmental
GhPHD22	GhPHD51	0.080	0.398	0.200	Yes	Segmental
GhPHD22	GhPHD65	0.007	0.028	0.240	Yes	Segmental
GhPHD22	GhPHD104	0.079	0.391	0.201	Yes	Segmental
GhPHD28	GhPHD77	0.085	0.284	0.298	Yes	Segmental
GhPHD28	GhPHD82	0.042	0.037	1.121	No	Segmental
GhPHD24	GhPHD78	0.003	0.039	0.086	Yes	Segmental
GhPHD25	GhPHD73	0.057	0.434	0.131	Yes	Segmental
GhPHD25	GhPHD79	0.016	0.017	0.982	Yes	Segmental
GhPHD26	GhPHD44	0.204	0.368	0.553	Yes	Segmental
GhPHD26	GhPHD98	0.186	0.344	0.541	Yes	Segmental
GhPHD29	GhPHD83	0.019	0.041	0.473	Yes	Segmental
GhPHD31	GhPHD85	0.011	0.031	0.356	Yes	Segmental
GhPHD32	GhPHD86	0.015	0.027	0.554	Yes	Segmental
GhPHD34	GhPHD88	0.006	0.021	0.288	Yes	Segmental
GhPHD36	GhPHD89	0.015	0.031	0.499	Yes	Segmental
GhPHD39	GhPHD92	0.014	0.040	0.339	Yes	Segmental
GhPHD40	GhPHD73	0.051	0.442	0.116	Yes	Segmental
GhPHD40	GhPHD93	0.002	0.054	0.035	Yes	Segmental
GhPHD41	GhPHD94	0.013	0.031	0.416	Yes	Segmental
GhPHD44	GhPHD98	0.022	0.052	0.413	Yes	Segmental
GhPHD46	GhPHD54	0.127	0.418	0.304	Yes	Segmental
GhPHD46	GhPHD100	0.014	0.055	0.256	Yes	Segmental
GhPHD46	GhPHD107	0.101	0.395	0.256	Yes	Segmental
GhPHD47	GhPHD67	0.231	0.450	0.514	Yes	Segmental
GhPHD47	GhPHD101	0.016	0.030	0.530	Yes	Segmental
GhPHD49	GhPHD102	0.006	0.035	0.163	Yes	Segmental
GhPHD50	GhPHD103	0.011	0.048	0.233	Yes	Segmental
GhPHD51	GhPHD65	0.079	0.389	0.202	Yes	Segmental
GhPHD51	GhPHD104	0.010	0.036	0.270	Yes	Segmental
GhPHD52	GhPHD80	0.038	0.528	0.072	Yes	Segmental
GhPHD53	GhPHD62	0.039	0.606	0.064	Yes	Segmental
GhPHD54	GhPHD100	0.140	0.416	0.336	Yes	Segmental
GhPHD54	GhPHD107	0.136	0.405	0.335	Yes	Segmental
GhPHD55	GhPHD106	0.023	0.045	0.497	Yes	Segmental
GhPHD56	GhPHD76	0.236	0.639	0.369	Yes	Segmental
GhPHD56	GhPHD108	0.007	0.051	0.132	Yes	Segmental
GhPHD60	GhPHD95	0.125	0.397	0.314	Yes	Segmental
GhPHD65	GhPHD104	0.078	0.386	0.203	Yes	Segmental
GhPHD67	GhPHD101	0.225	0.456	0.492	Yes	Segmental
GhPHD71	GhPHD81	0.075	0.369	0.203	Yes	Segmental
GhPHD73	GhPHD79	0.054	0.486	0.111	Yes	Segmental
GhPHD73	GhPHD93	0.053	0.419	0.127	Yes	Segmental
GhPHD76	GhPHD108	0.237	0.630	0.376	Yes	Segmental
GhPHD91	GhPHD38	0.016	0.052	0.298	Yes	Segmental
GhPHD100	GhPHD107	0.104	0.379	0.276	Yes	Segmental
GhPHD2	GhPHD3	0.035	0.122	0.289	Yes	Tandem
GhPHD32	GhPHD33	0.031	0.076	0.407	Yes	Tandem
GhPHD58	GhPHD59	0.029	0.138	0.212	Yes	Tandem
GhPHD86	GhPHD87	0.027	0.062	0.428	Yes	Tandem

### Gene structure and conserved motifs analysis

**Fig. 2:**
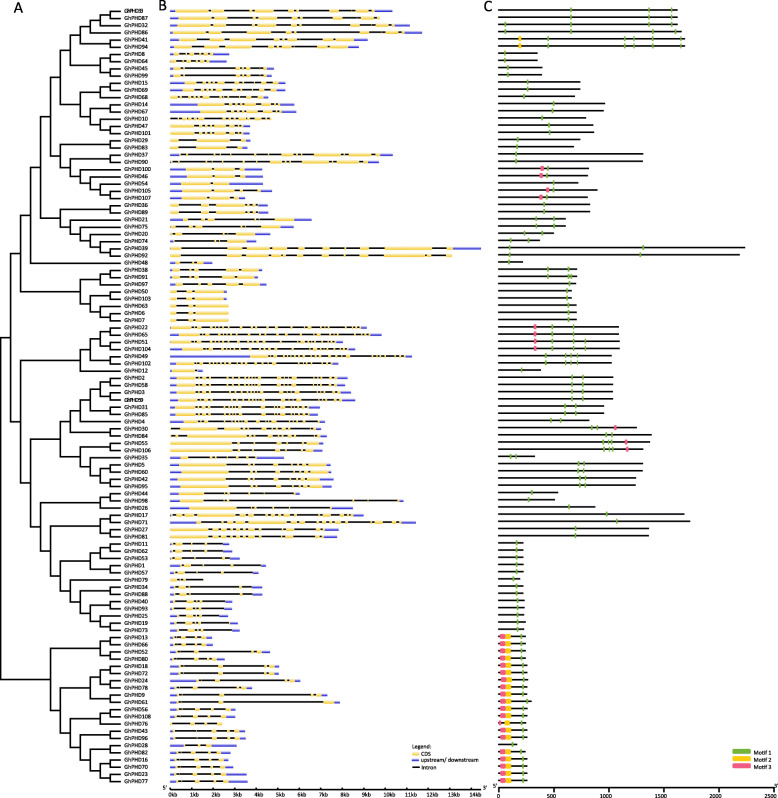
Phylogenetic tree, gene structure, and conserved motif analysis of GhPHD proteins. **a** An unrooted phylogenetic tree was generated in MEGA 6.0 by neighbor-joining (NJ) method. **b** Exon-intron structure of *GhPHD* genes. The yellow boxes represent exons, black lines represent introns, and blue boxes represent the upstream/downstream UTRs. The sizes of exon and intron can be estimated using the scale bar at the bottom. **c** Motifs distribution of GhPHD proteins and different motif boxes are represented in different colors (motif 1 to 3). Motif 1 is the PHD domain

To better understand the similarity and diversity of GhPHD proteins in upland cotton, we analyzed the phylogenetic tree, exon-intron structure, and conserved motif. Phylogenetic tree grouped GhPHD proteins according to protein homology, conserved gene structure, and motif distribution (Fig. 2). *GhPHD49* shows the longest genomic sequence with 26 exons, while *GhPHD12* displays the shortest genomic sequence with only two exons (Fig. 2 and Table S3). Furthermore, a total of three motifs are identified in all GhPHD proteins, and all GhPHD proteins have a typical PHD domain (i.e., motif 1). Phylogenetic tree showed that 21 GhPHD proteins are clustered in a clade. Except for GhPHD28, all other GhPHD proteins contain three motifs with similar gene structure and motif distribution (Fig. 2).

Protein sequence alignment shows that GhPHD proteins have a typical Cys4-His-Cys3 motif, which consists of about 60 amino acids and is accompanied by nine conserved amino acid residues (Fig. S2). The conserved histidine (H) is separated from the fourth conserved cysteine (C) by four amino acids and two amino acids from subsequent conserved cysteine (C) residue. The third and fourth conserved cysteine (C) before histidine (H) are separated by one or two amino acids, but the interval number between other conserved amino acids is uncertain. However, GhPHD17, GhPHD27, GhPHD71, and GhPHD81 exhibit maximum homology, but show less conserved PHD domain (Fig. 2 and Fig. S2).

### *Cis*-acting element analysis

**Fig. 3:**
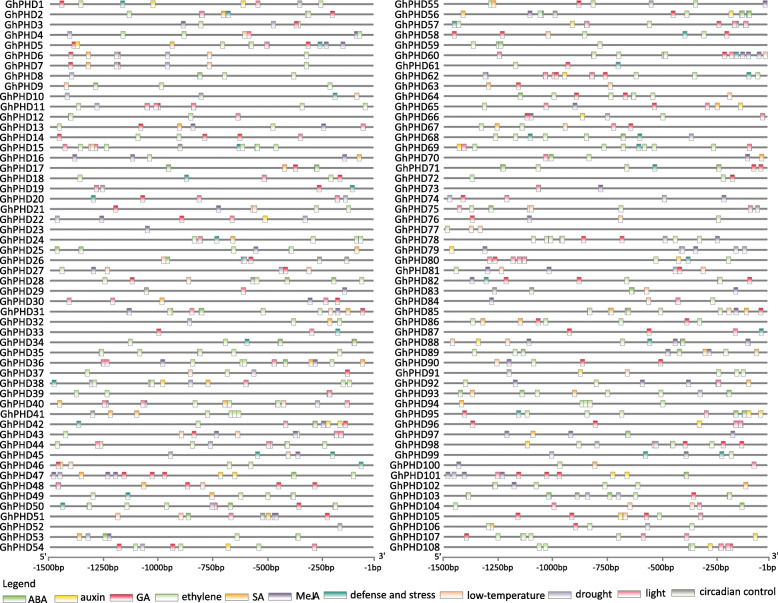
Distribution of stress-related and phytohormone-related *cis*-acting elements in the promoter regions of *GhPHD* genes. The locations of *cis*-acting elements were confirmed using PlantCARE database. Different *cis*-acting elements were represented by different color boxes

Many studies have showed that *PHD* genes are involved in various stress responses [30, 31, 37]. To elucidate the putative function of *GhPHDs* under different stresses, we first identified the *cis*-acting elements in the promoter region that respond to stresses and phytohormones. We identified many *cis*-acting elements that respond to ABA (ABRE), auxin (TGA and AuxRR-core), GA (TATC-box, P-box, CARE, and GARE), ethylene (ERE), SA (TCA), and MeJA (CGTCA). These results indicated that a total of 85 *GhPHD* genes are responsive to ethylene, followed by ABA, GA, and MeJA. 73 *GhPHD* genes have *cis*-acting elements that respond to three or more phytohormones. Interestingly, the promoters of *GhPHD5*, *GhPHD47*, *GhPHD56*, and *GhPHD65* genes contain *cis*-elements that respond to the above six phytohormones. In addition, we found that many abiotic stresses response elements (TC-rich repeat, MBS, and LTR), circadian control elements, and light-responsive elements (G-box) are also present in the promoters of various *GhPHD* genes (Fig. 3 and Table S5). These results indicated that *GhPHD* genes may participate in various signal transduction pathways, such as phytohormones, light response, and abiotic stresses, and play important roles in regulating plant growth and development.

### Tissue-specific expression pattern of *GhPHD* genes

**Fig. 4:**
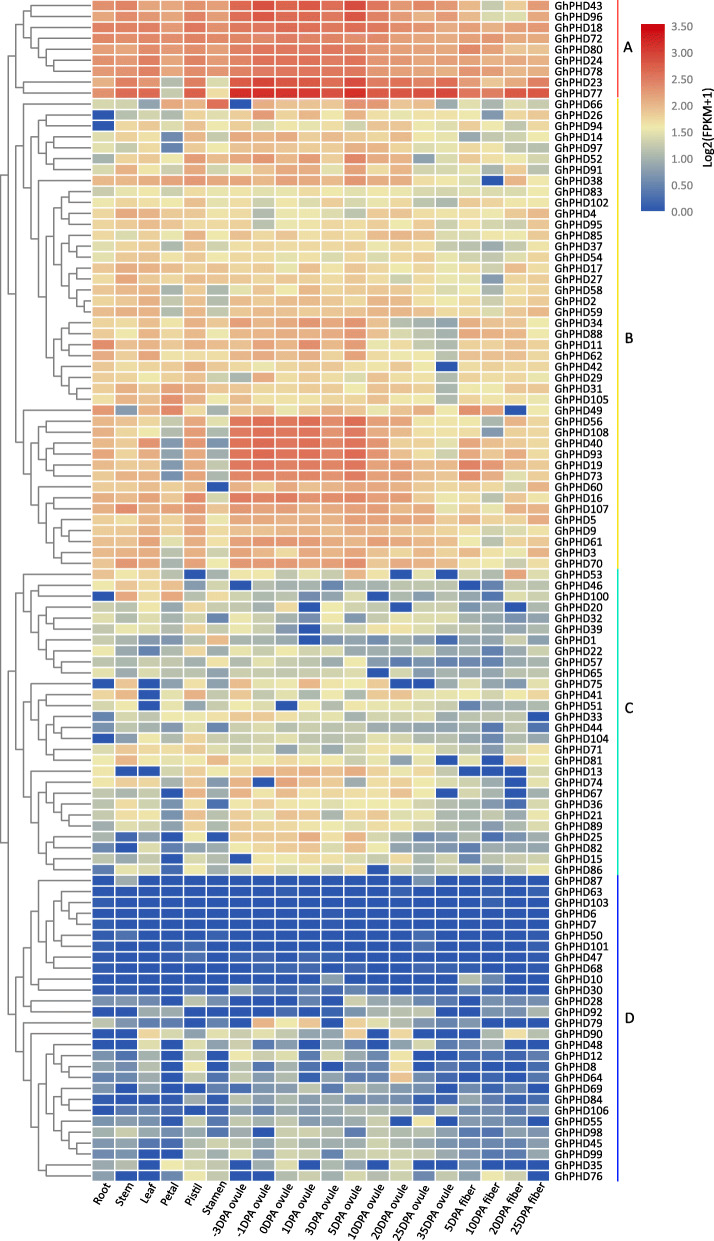
Tissue-specific expression patterns of *GhPHD* genes in upland cotton. A heatmap indicates the clustering of 108 *GhPHD* genes in eight tissues (shown at the bottom). DPA is days post anthesis. Gene names are shown on the right. Scale bars at the top show Log_2_ (FPKM+ 1) values of each gene

To predict the physiological functions of *GhPHD* genes in cotton growth and development, we used the online transcriptome data to analyze the tissue-specific expression profile of *GhPHD* genes in different tissues such as root, stem, leaf, petal, stamen, pistil, ovule, and fiber. According to the expression features and hierarchical clustering (Fig. 4), *GhPHD* genes are mainly clustered into four groups (A-D). The nine *GhPHD* genes in group A are highly expressed in all tissues, indicating that they may play important roles in plant growth and development. In particular, *GhPHD23* and *GhPHD77* show maximum expression levels in ovule and fiber tissues, demonstrating that these two genes may be involved in the development of ovule and fiber. Further, 43 *GhPHDs* in group B show lower expression levels in all tissues, while six *GhPHD* genes (*GhPHD56*, *GhPHD108*, *GhPHD40*, *GhPHD93*, *GhPHD19*, and *GhPHD73*) are predominantly expressed in the early stage of ovule development, indicating that they may play important roles in ovule and seed development. Moreover, *GhPHD* genes in group C show higher expression levels in ovule. However, *GhPHD* genes in group D show poor expression in all observed tissues. These results indicated that *GhPHDs* may be involved in regulating cotton growth and development, especially in the development of ovule and fiber.

### Identification of stress-related *PHD* genes in upland cotton

**Fig. 5:**
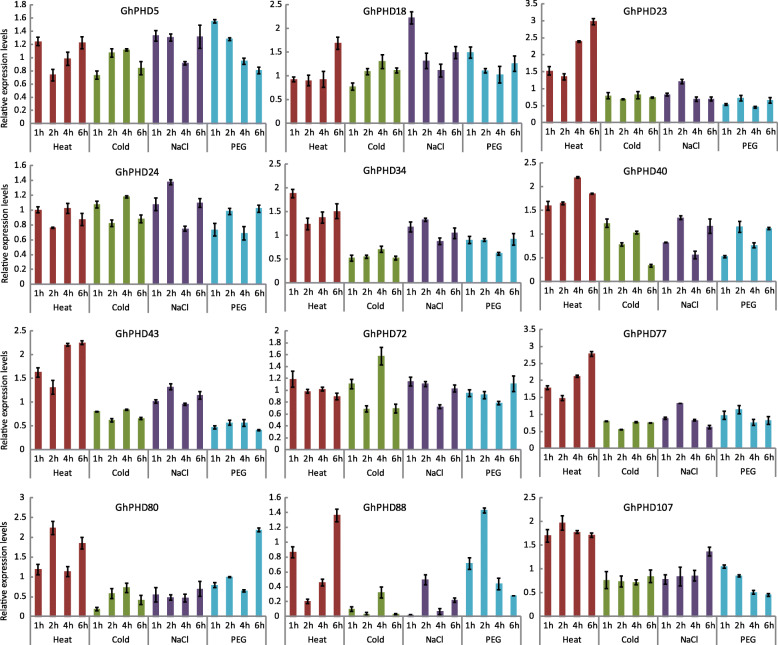
The relative expression levels of 12 *GhPHD* genes under heat, cold, salt, and drought treatment. The relative expression levels were estimated by RT-qPCR. The error bars represent the standard deviations of three experiments

Analysis of the transcriptome data showed that 66 *GhPHD* genes have higher expression levels under heat, cold, salt, and drought treatments (Fig. S3). In order to further estimate the responses of *GhPHDs* under abiotic stresses, we treated four-week-old cotton seedlings with heat, cold, salt, and drought, and observed the relative expression level of 12 *GhPHD* genes (Fig. 5). The relative expression level of *GhPHD18* is up-regulated under all stresses, indicating that *GhPHD18* may be involved in multiple stresses response mechanisms. *GhPHD23* is up-regulated only under heat treatment, indicating that *GhPHD23* responds positively to heat stimuli. Further, *GhPHD34*, *GhPHD40*, and *GhPHD43* are up-regulated after heat and salt treatment, while *GhPHD80* and *GhPHD88* are up-regulated after heat and drought tolerance at various time points. In addition, we found that *GhPHD5* is up-regulated against salt and drought, while *GhPHD72* and *GhPHD107* are up-regulated against salt and heat, respectively. These results indicated that *GhPHD* genes may be involved in abiotic stress to improve plant tolerance in adverse environments.

### Identification of *GhPHD* genes in response to phytohormones

**Fig. 6:**
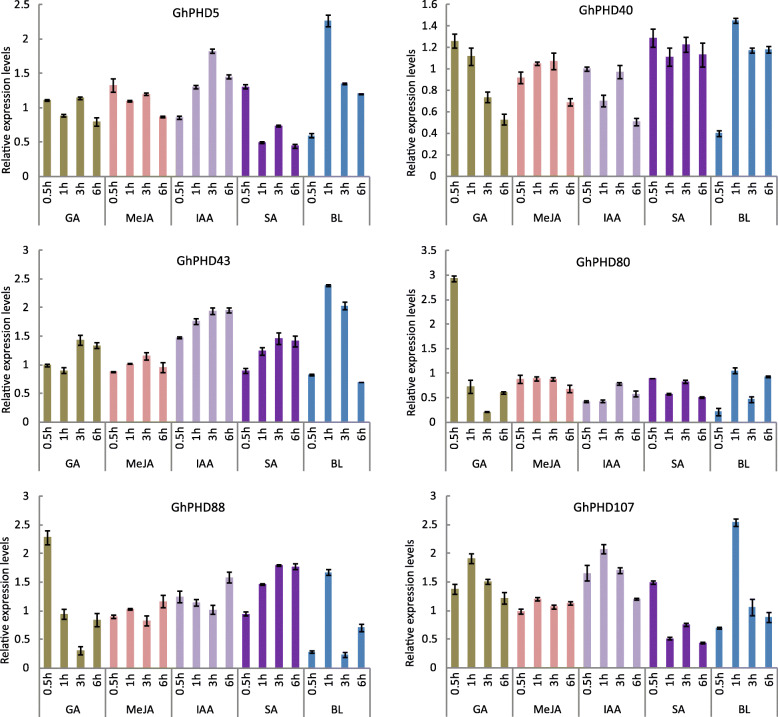
The relative expression levels of six *GhPHD* genes under GA, MeJA, IAA, SA, and BL treatment. The relative expression levels were estimated by RT-qPCR. The error bars show the standard deviation of three biological replicates

To further determine whether *GhPHD* genes respond to phytohormones, we treated four-week-old cotton seedlings with GA, MeJA, IAA, SA, and BL, and identified changes in the relative expression of *GhPHD* genes (Fig. 6). The relative expression level of *GhPHD5* increases significantly after MeJA, IAA, and BL treatment. While *GhPHD5* shows higher expression after 0.5 h after SA treatment indicating that *GhPHD5* may respond to multiple phytohormones signal transduction pathway, which is consistent with the fact that *GhPHD5* promoter contains *cis*-acting elements related to multiple phytohormones. *GhPHD40* is significantly up-regulated under SA treatment, indicating that *GhPHD40* responds positively to SA signal. Similarly, *GhPHD43* is significantly up-regulated under all phytohormone treatments, especially under BL. The relative expression levels of *GhPHD80* and *GhPHD88* reach at peak after 0.5 h of GA treatment. The relative expression level of *GhPHD88* increases gradually under SA treatment. Moreover, *GhPHD107* expression significantly increases to the maximum level after 1 h of GA, IAA, and BL treatment. These results indicated that *GhPHD* genes are involved in regulating multiple phytohormone signal transduction pathways.

### Co-expression network with functional modules for *G. hirsutum* and *G. arboreum*

**Fig. 7:**
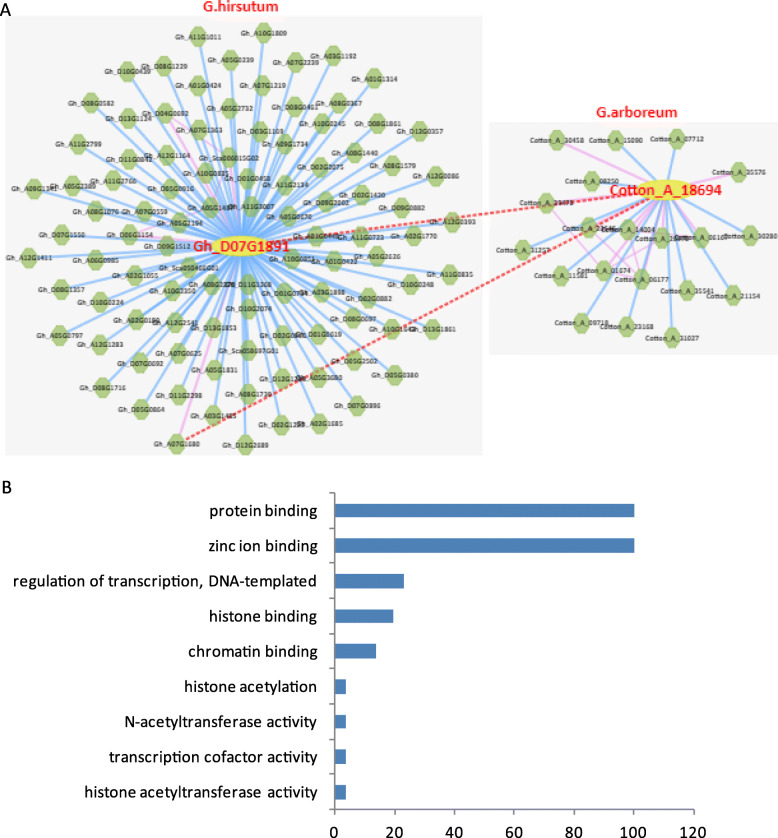
Co-expression networks analysis of *GhPHD88* and GO enrichment analysis of 108 *GhPHDs*. **a** Co-expression network analysis of *GhPHD88* with functional modules for *G. hirsutum* and *G. arboreum*. Yellow and green colour indicates that query protein and interaction proteins, respectively. There are four interaction lines, red lines indicated ortholog gene pairs in *G. hirsutum* and *G. arboreum*; pink lines and blue lines indicate proteins own interaction and positive/negative co-expression relationship with target protein; orange lines indicate proteins own interaction and protein-protein relationship with target protein. **b** GO enrichment analysis of all *GhPHD* genes

Gene co-expression network analysis is a network diagram constructed on the basis of similarity of gene expression data, reflecting the relationship of expression regulation between genes [45]. We analyzed the co-expression network of *GhPHD* genes using ccNET software, and predicted many co-expressed genes and interaction proteins (Table S6). Among these, *GhPHD5* is positively co-expressed with a plant-specific DNA ligase, which is related to seed germination and DNA repair. In addition, *GhPHD5* is also positively co-expressed with SLOMO protein, which is a F-box protein required for auxin homeostasis and the normal timing of lateral organ initiation at the shoot meristem [46] illustrating that *GhPHD5* may be involved in the regulation of auxin signal transduction pathway, and mediates seed germination and organ formation to regulate plant growth and development. Similarly, *GhPHD18* interacts with highly hydrophilic proteins that regulate FLC (Flowering locus C) expression [47] and shows positively co-expressed with SHAGGY-related kinases involved in meristem organization, indicating that *GhPHD18* may affect the flowering time of meristem. Further, *GhPHD34* negatively co-expressed with ERF (Ethylene response factor) subfamily *B-1*, participating in ethylene signaling pathway and responding to abiotic stresses. *GhPHD107* positively co-expressed with *ARF-GAP* and *ERF* genes, and may be involved in the signal pathways of auxin and ethylene. More interestingly, we predicted many proteins that interact with *GhPHD88*, such as leucine-rich repeat protein kinase (LRRK), late embryogenesis abundant (LEA) protein, AP2/B3 transcription factor, R2R3 factor, DREB subfamily A-2, cellulose synthase, gibberellin-regulated family protein (GRP), and ethylene response factor (ERF) (Fig. 7a and Table S6), suggesting that *GhPHD88* may be involved in many physiological processes such as plant growth and development, phytohormone signal transduction, and stress response. Further, Gene Ontology (GO) analysis of *GhPHDs* indicated that protein binding and zinc ion binding are the most abundant functional terms (Fig. 7b), which is consistent with the existing results that the cysteine residues exhibit high affinity for zinc ions (Zn^2+^), and Zn^2+^-cysteine complexes are key medium for protein structure, catalysis, and regulation [48].

In summary, *GhPHDs* were involved in regulating cotton growth and development, especially ovule and fiber development. Further, *GhPHDs* not only respond to multiple phytohormones signal transduction pathways, but also improve cotton’s tolerance to adverse environments such as heat, salt, and drought. Particularly, *GhPHD5*, *GhPHD80*, *GhPHD88* are prominent in their responses. Combining the predicted results of co-expressed genes and interacting proteins, we inferred that phytohormones could improve plant tolerance to abiotic stresses through *GhPHD* genes and their cofactors, but their regulatory mechanism and interaction network still need further research.

## Discussion

### Phylogenetic analysis and duplication

Phylogenetic tree was used to analyze the evolutionary relationship between PHD proteins in cotton, rice, and *Arabidopsis*. A total of 297 PHD proteins were divided into five groups (A-E). The relationship between cotton PHD proteins and AtPHD proteins was closer than that of OsPHD proteins, which is consistent with the evolutionary relationship between cotton, *Arabidopsis*, and rice. Although the *G. arboreum* genome is about twice that of the *G. raimondii* genome, however, more GrPHD proteins were identified than GaPHD proteins. Most PHD proteins from two diploids and one allotetraploid were closely distributed in phylogenetic tree, which is coherent with the fact that upland cotton evolved from the hybridization of A and D genomes [49].

We identified 108 GhPHD proteins in the *G. hirsutum* genome, which are more than previously identified PHD protein family members in *Arabidopsis*, maize, potato, and pear [30, 39, 40]. The main reason for the more number of *GhPHDs* is that upland cotton underwent polyploidization and promoted gene duplication. Upland cotton is an allotetraploid cotton produced by the hybridization between *G. arboreum* (A_2_ genome) and *G. raimondii* (D_5_ genome) [49]. The At and Dt subgenome donors of upland cotton are orthologous relatives and share the same number of ortholog genes, resulting in the duplication and doubling of *GhPHD* genes in upland cotton. Therefore, the sum total of *GaPHD* genes and *GrPHD* genes was approximately equal to the number of *GhPHD* genes. Previous studies have reported that gene duplication, including whole genome duplication, segment duplication, tandem duplication, and transposition events was the main reason for gene family expansion [50, 51]. In our study, a total of 77 duplicated gene pairs were identified in *GhPHD* family, including 73 segmental duplicated pairs and four tandem duplicated pairs (Table 2). The *Ka/Ks* values of most *GhPHD* duplication gene pairs was less than 1, which indicated that *GhPHD* family experienced strong purification selection pressure. Purification selection dominated the expansion of *GhPHD* genes, eliminated deleterious loss-of-function mutations at both duplicated loci, increased fixation, and retained the function of the new duplicated genes [52].

### Conserved amino acid residues, protein motifs, and gene structure analysis

Conserved amino acid residues analysis showed that GhPHD domain was highly conserved during the process of evolution. The amino terminus of GhPHD domain contained the Cys4-His-Cys3 zinc finger motif composed of 50 to 80 amino acids with the regular arrangement of cysteine residues, an important medium for zinc ion binding and protein structure [48]. In addition, a total of three motifs were identified in GhPHD proteins and the motif distribution was relatively conservative, indicating that GhPHD proteins may play different physiological functions, and the subtle differences between GhPHD proteins in different clade may be related to cotton growth, development, and stress tolerance.

Gene structure may be determined by the insertion/deletion events and is an important parameter to predict gene evolution and new function generation [53]. Gene structure analysis indicated that the duplication genes showed similar gene structures with varied intron length indicating that the intron length may play major roles in the functional diversification of *GhPHD* genes. In this study, we found that the intron number varies from 1 to 25, but most *GhPHD* genes contained 2 to 11 introns which supported the previous research that cotton is a new evolution species that experience a decrease in the number of introns during the early stages of evolution [54].

### *GhPHD* genes expression in tissues, abiotic and phytohormone stresses

Many studies demonstrated that PHD proteins are the main mediators of transcriptional regulation during plant developmental processes such as meiosis and postmeiotic events [55], germination [34], pollen maturation [56], flowering time [57], embryo meristem initiation, and root development [55, 58]. Gene’s expression profiles showed that *GhPHDs* may play important regulatory roles in cotton growth and development, especially during the development of ovule and fiber. In addition, we have also identified some *GhPHD* genes that respond to abiotic stress and phytohormones in upland cotton. The analysis of *cis*-acting elements and seedlings treatment experiments indicated that *GhPHD* genes may respond to abiotic stress and participate in the signal transduction of phytohormones. For example, *GhPHD* genes (*GhPHD5*, *GhPHD40*, *GhPHD43*, *GhPHD80*, and *GhPHD88*) respond positively to heat, salt, and drought and they may be important genetic materials for improving plant tolerance under adverse environments.

Research reports indicated that phytohormones may regulate the response to abiotic stress in plants. Auxin response factors (ARFs) are a type of transcription factors that regulate the expression of auxin-responsive genes [59, 60]. The significant up-regulation of the transcription level of ARFs under stress indicates that they are potential mediators for plants to respond to adverse environments [61, 62]. Ethylene response factors belong to the ERF subfamily of the AP2/ARF transcription factor family, and are widely involved in plant development, phytohormones response, disease resistance, and adversity response [63, 64]. In this study, co-expression network analysis indicated that *GhPHD* genes may improve plant tolerance to abiotic stresses by phytohormone signaling pathways. For instance, *GhPHD5* may improve tolerance to heat, salt, and drought by regulating auxin homeostasis. Similarly, *GhPHD34* and *GhPHD107* may be involved in auxin and ethylene signal transduction pathways to improve heat tolerance and promote growth and development. *GhPHD88* regulates the signal transduction of various phytohormones and abiotic stresses, and promotes growth and development. Although *GhPHDs* are indispensable in the course of life, the physiological functions of *GhPHDs* in crosstalk between abiotic stress and phytohormone need further study.

## Conclusions

In this study, a total of 297 PHD proteins were identified in total five plant species including *G. hirsutum*, *G. arboreum*, *G. raimondii*, rice, and *Arabidopsis*. The PHD proteins were divided into five groups based on the phylogenetic analysis. Segmental duplication events were the main contributors toward the expansion of *GhPHD* gene family in upland cotton. Moreover, duplicated gene pairs of *GhPHD* gene family might have experienced functional divergence, since their expression patterns were different in different tissues. Tissues specific expression patterns indicated that *GhPHDs* are very important for growth and development, especially ovule and fiber development. The phytohormones and stresses treatment and co-expression network analysis showed that *GhPHDs* may improve the tolerance to adverse environments by phytohormones signal transduction pathway. Taken together, our study provides key basic knowledge to understand the functional mechanisms of cotton growth and development, as well as candidate genes for cotton breeding resistant to abiotic stresses and phytohormone stimulation.

## Methods

### Sequence retrieval, multiple sequence alignment, and phylogenetic analysis

The genome sequence and information of cotton (*G. hirsutum*, *G. raimondii*, and *G. arboreum*) were acquired from the CottonFGD (https://cottonfgd.org/) [65]. HMMER (https://www.ebi.ac.uk/Tools/hmmer/) software with default parameters was used to search for the corresponding protein sequences, and used the conserved PHD domain sequence as a query. We used BLAST program to further identify PHD sequences based on homology. The conserved domain of PHD proteins was predicted by Pfam [66] and SMART [67] software. Multiple sequence alignment of PHD proteins were performed using Clustal X [68]. MEGA 6.0 [69] was used to construct phylogenetic trees, using the neighbor-joining (NJ) algorithm with default parameters and 1000 bootstrap replicates. The molecular weight (MW), isoelectric point (pI), and GRAVY value of GhPHD proteins were predicted using ExPASy [70], and the subcellular localization of GhPHD proteins was predicted by the CELLO v2.5 server [71].

### Chromosomal location, gene structure, and conserved motif

The positional information of *GhPHD* genes was obtained from the General Feature Format (GFF) file downloaded from the CottonFGD website [65]. *GhPHDs* were mapped on the chromosome using MapInspect (https://mapinspect.software.informer.com/). For the exon-intron structural analysis of *GhPHD* genes, the coding sequences were used to align their genomic DNA sequences and the structure diagram was drawn using the online Gene Structure Display Server (GSDS 2.0) program [72]. Conserved motifs of GhPHD proteins were investigated using the online toolkit Multiple Expectation maximization for Motif Elicitation (MEME 5.0.5) [73]. The optimized parameters of MEME are as follows: the number of repetitions, any; the maximum number of motifs, 50; and the optimum width of each motif, between 6 and 300 residues, and retaining only motifs associated with an E value < e^− 5^. The identified protein motifs were further annotated with TBtools [74].

### Identification of *cis*-acting elements and gene expression pattern

The 1500 bp promoter sequence before the transcription start site of *GhPHD* genes were downloaded from the CottonFGD website [65]. The *cis*-acting elements in the *GhPHD* promoter regions were predicted using the Plant Cis-Acting Regulatory Element website [75]. The tissue expression patterns of *GhPHD* genes were analyzed using the online cotton transcriptome data, and heatmap was drawn by TBtools [74]. The transcriptome data of root, stem, leaf, petal, stamen, pistil, ovule (− 3, − 1, 0, 1, 3, 5, 10, 20, 25, 35 DPA) and fiber (5, 10, 20, 25 DPA) was used in this study. The ccNET software [76] was used to analyze the gene co-expression network relationship.

### Plant material, abiotic stresses and phytohormones treatment

Upland cotton ZM24 is a short-season cotton variety selected by the Cotton Research Institute of Chinese Academy of Agricultural Sciences. Firstly, ZM24 seeds were pre-germinated in the conical flask filled with water at room temperature for 48 h. Pre-germinated seeds were then transferred to the liquid medium with a cultivation temperature of 30 °C, a photoperiod of 16 h light and 8 h dark. Four-week-old cotton seedlings were treated with brassinolide (BL, 10 μM), gibberellin (GA, 100 μM), indole-3-acetic acid (IAA, 100 μM), salicylic acid (SA, 10 μM), and methyl jasmonate (MeJA, 10 μM) for 0.5, 1, 3, and 6 h. Similarly, four-week-old cotton seedlings were treated with heat (38 °C), cold (4 °C), NaCl (200 mM), and polyethylene glycol (PEG) (20% mass fraction) for 1, 2, 4, and 6 h. In the experiment, the untreated sample was used as the control group. The collected leaves were immediately frozen in liquid nitrogen and stored at − 80 °C for RNA extraction and RT-qPCR analysis. For abiotic stresses and phytohormones treatment, a total of 20 cotton seedlings were used for each treatment and three biological replicates were performed for each experiment.

### RNA extraction and RT-qPCR analysis

Total RNA of the collected cotton leaves was extracted using the RNAprep Pure Plant Kit (Polysaccharides & Polyphenolics-rich) (TianGen, Beijing, China). In order to synthesize the first-strand cDNA, the EasyScript All-in-One First-strand cDNA synthesis SuperMix for RT-qPCR kit (TransGen, Beijing, China) was used in accordance with the manufacturer’s protocol and the cDNA was used as template for subsequent RT-qPCR reaction. RT-qPCR was performed using TransStart Top Green qPCR SuperMix (TransGen, Beijing, China) in LightCycler 480 (Roche, Basel, Switzerland). Each PCR reaction was performed in triplicate, and three biological replicates were quantified. *GhHistone 3* (GenBank accession no. AF024716) was used as an internal control [77]. The relative expression level was calculated as described previously [78]. The primers used for RT-qPCR analysis were listed in Table S7. For statistical analysis, the RT-qPCR data was considered as normal distribution and we conducted a two-tailed Student’s *t*-test in Microsoft Excel 2007.

## Supplementary Information

**Additional file 1: Fig. S1**. Chromosomal location of *GhPHD* genes on 26 chromosomes in *G. hirsutum*. The chromosome numbers were shown on the top of each chromosome. The scale bar indicated the length in megabases (Mb)

**Additional file 2: Fig. S2**. Alignment results from the conserved domain of 108 GhPHD proteins and PHD motifs with a typical C_4_HC_3_ model

**Additional file 3: Fig. S3**. Expression profiles of *GhPHD* genes under cold, hot, salt, and drought. The expression characteristics of 108 *GhPHD* genes under four stress treatments were investigated using available transcriptomic data. 1 h, 3 h, 6 h, and 12 h indicate hours after different stress treatments. Gene names and the subfamilies are shown on the right. Blocks with colors represent the relative expression levels of *GhPHDs*

**Additional file 4: Table S1**. The PHD members from *G. hirsutum*, *G. raimondii*, *G. arboreum*, *A. thaliana*, and *O. sativa*

**Additional file 5: Table S2**. Chromosomal location and gene annotation of *GhPHD* genes in *G. hirsutum*

**Additional file 6: Table S3**. Transcript-features of 108 *GhPHD* genes

**Additional file 7: Table S4**. Distribution of major stress-related and phytohormone-related *cis*-acing elements in the promoter regions of *GhPHD* genes

**Additional file 8: Table S5**. Number of *cis*-acting elements in the promoters of *GhPHD* genes

**Additional file 9: Table S6**. Co-expression network analysis results

**Additional file 10: Table S7**. Primers for RT-qPCR in this study

## Data Availability

The data used or analyzed during the current study has been included in this article and additional materials. The genome sequence and annotation datasets that supported the findings of this study are available in: *A. thaliana*: https://www.arabidopsis.org *O. sativa*: http://plants.ensembl.org/index.html *G. hirsutum*, *G. arboreum*, and *G. raimondii*: https://cottonfgd.org/

## Abbreviations

Gh: *Gossypium hirsutum*
Ga: *Gossypium arboreum*;
Gr: *Gossypium raimondii*
Os: *Oryza sativa*
At: *Arabidopsis thaliana*
DPA: Day post-anthesis
PHD: Plant homeodomain
NJ: Neighbor-joining
ARF: Auxin response factor
MMD1: Male meiocyte death 1
VIM1: Variant in methylation 1
VIN3: Vernalization insensitive 3
GSR1: Germostatin resistance locus 1
BR: Brassinosteroide
BL: Brassinolide
GA: Gibberellin
IAA: Indole-3-acetic acid
SA: Salicylic acid
MeJA: Methyl jasmonate
ABA: Abscisic acid
ET: Ethylene
CK: Cytokinin
PEG: Polyethylene glycol
RT-qPCR: Quantitative real-time polymerase chain reaction
GO: Gene Ontology
WGD: Whole genome duplication
FLC: Flowering locus C
ERF: Ethylene response factor
GRP: Gibberellin-regulated family protein
LEA: Late embryogenesis abundant
LRRK: Leucine-rich repeat protein kinase
