# Supplementary figures and images for: Response of phytohormone mediated plant homeodomain (PHD) family to abiotic stress in upland cotton (Gossypium hirsutum spp.)
Source: BMC Plant Biol. 2021 Jan 6;21:13. doi: 10.1186/s12870-020-02787-5 (PMC7788912; doi:10.1186/s12870-020-02787-5)

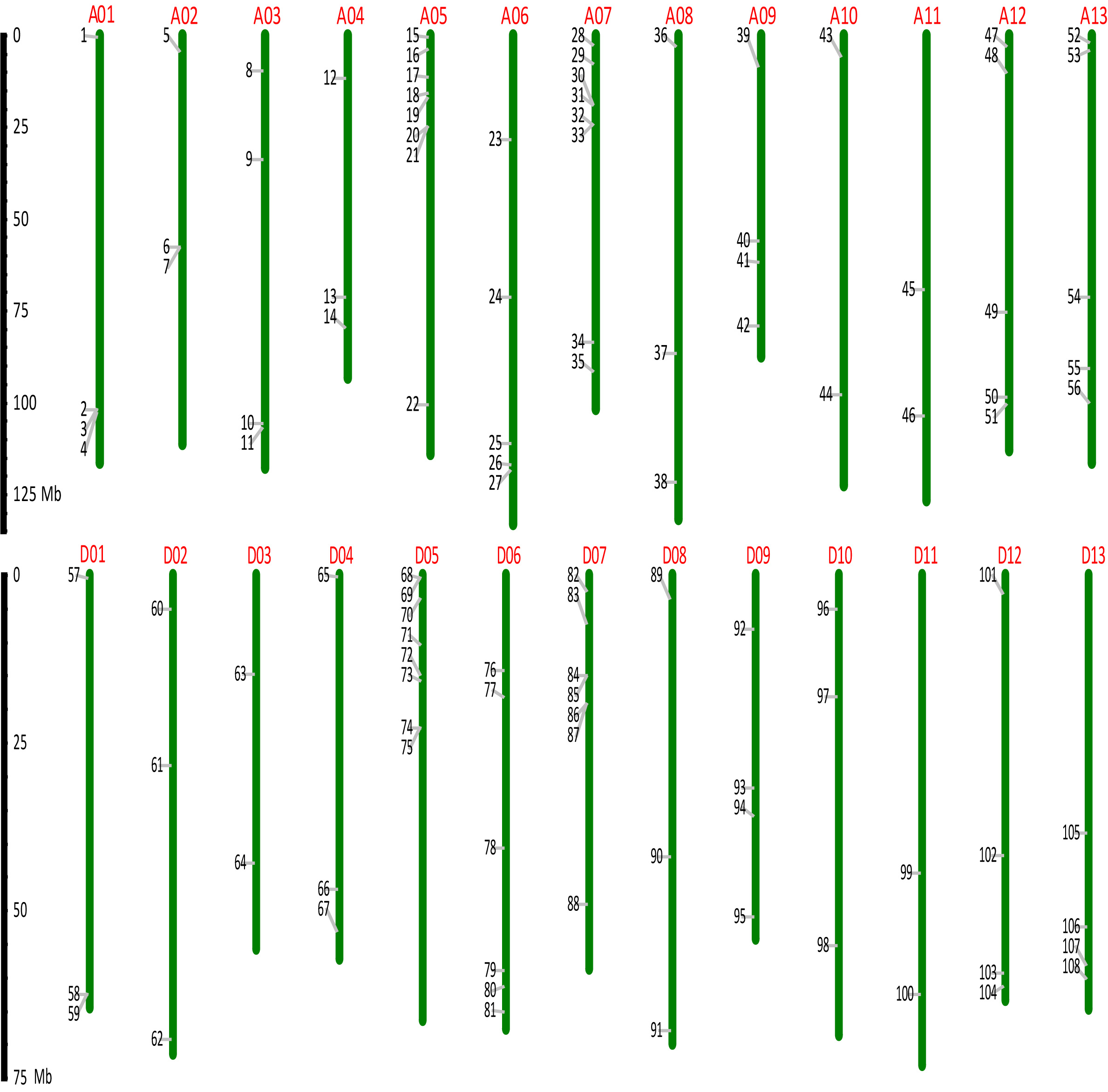

Supplement: Supplementary file 1 — Additional file 1: Fig. S1. Chromosomal location of GhPHD genes on 26 chromosomes in G. hirsutum. The chromosome numbers were shown on the top of each chromosome. The scale bar indicated the length in megabases (Mb) [file 12870_2020_2787_MOESM1_ESM.jpg]

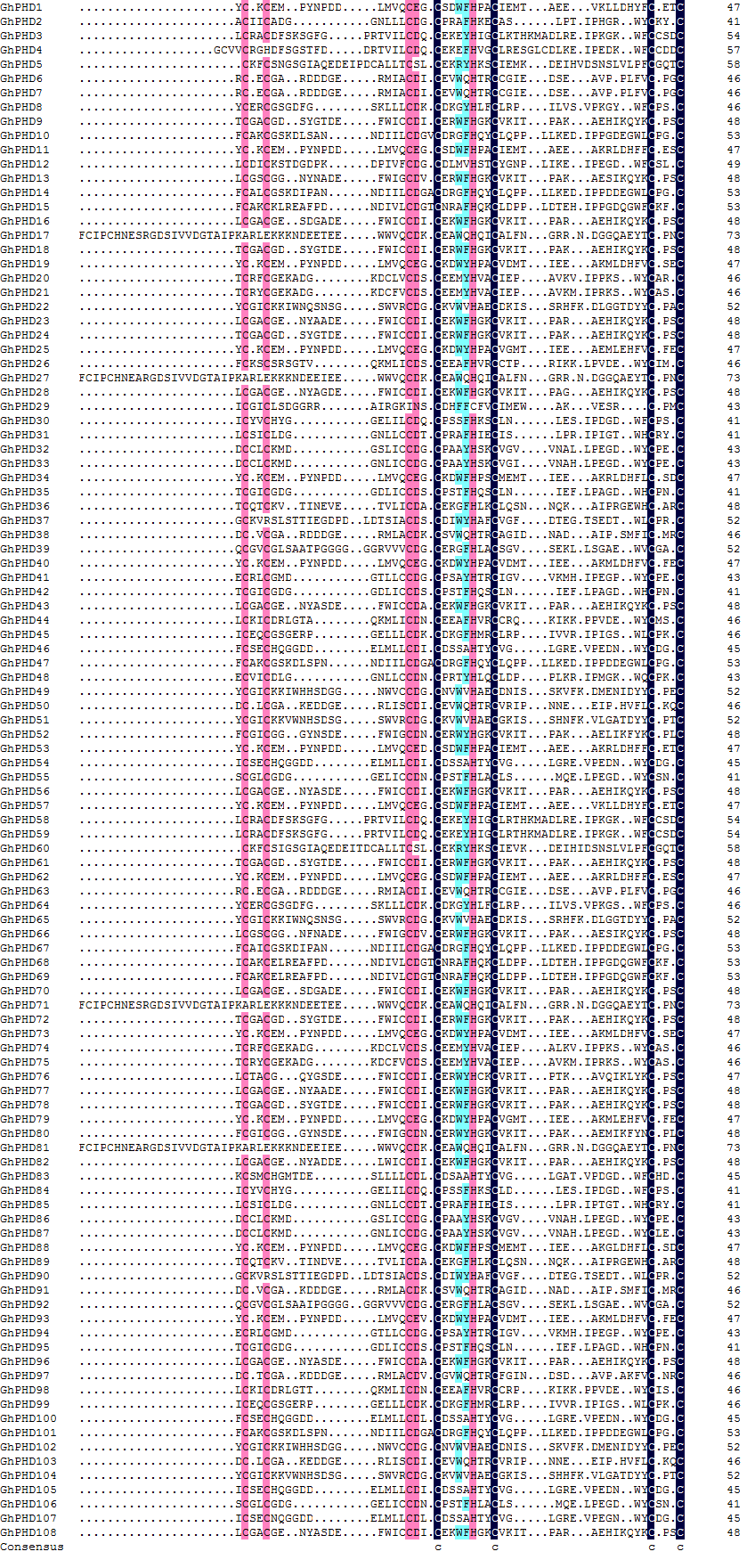

Supplement: Supplementary file 2 — Additional file 2: Fig. S2. Alignment results from the conserved domain of 108 GhPHD proteins and PHD motifs with a typical C4HC3 model [file 12870_2020_2787_MOESM2_ESM.jpg]

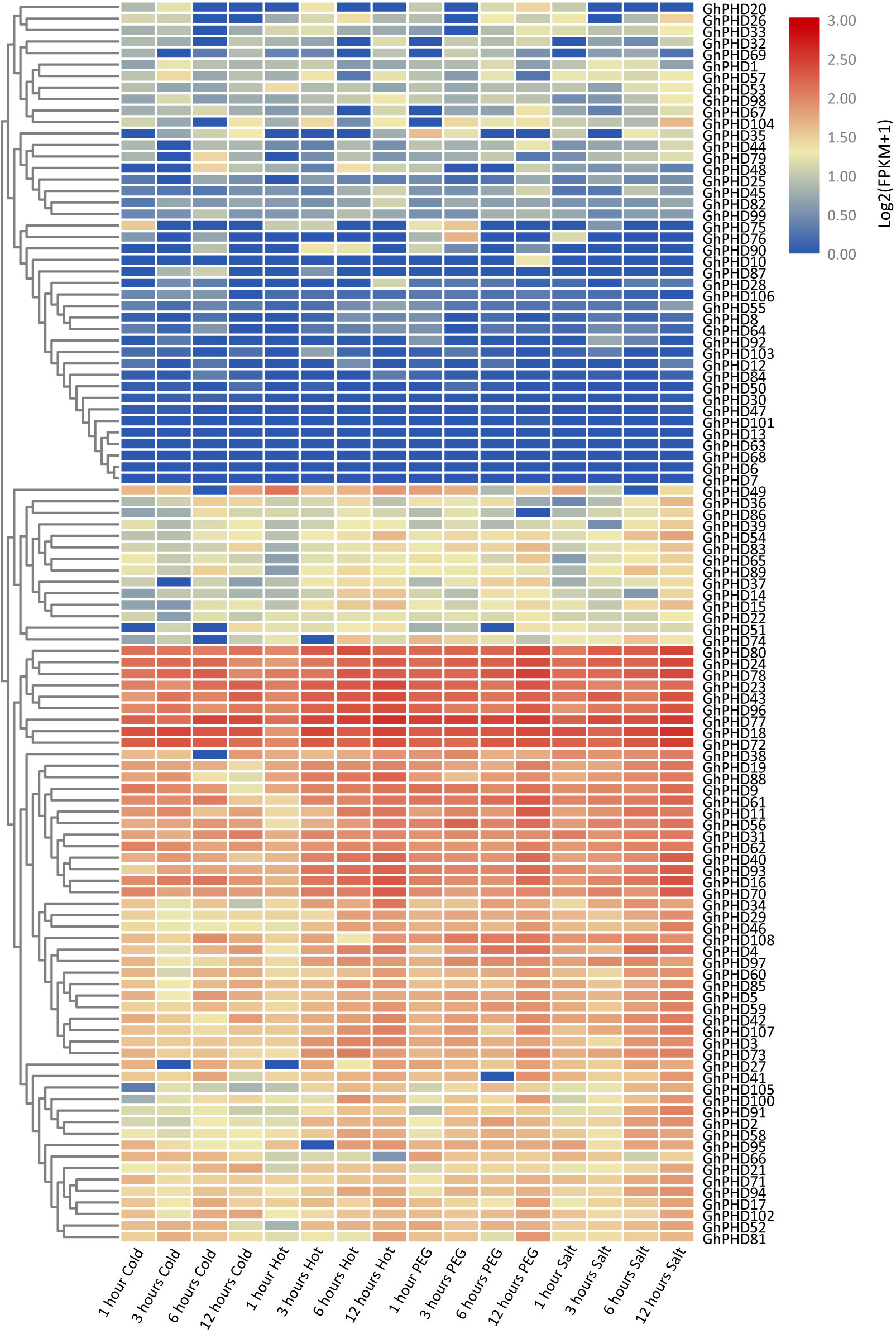

Supplement: Supplementary file 3 — Additional file 3: Fig. S3. Expression profiles of GhPHD genes under cold, hot, salt, and drought. The expression characteristics of 108 GhPHD genes under four stress treatments were investigated using available transcriptomic data. 1 h, 3 h, 6 h, and 12 h indicate hours after different stress treatments. Gene names and the subfamilies are shown on the right. Blocks with colors represent the relative expression levels of GhPHDs [file 12870_2020_2787_MOESM3_ESM.jpg]
