# Supplementary material for: Response of phytohormone mediated plant homeodomain (PHD) family to abiotic stress in upland cotton (Gossypium hirsutum spp.)
Source: BMC Plant Biol. 2021 Jan 6;21:13. doi: 10.1186/s12870-020-02787-5 (PMC7788912; doi:10.1186/s12870-020-02787-5)
Supplement: Supplementary file 5 — Additional file 5: Table S2. Chromosomal location and gene annotation of GhPHD genes in G. hirsutum [file 12870_2020_2787_MOESM5_ESM.docx]

**Table S2.** Chromosomal location and gene annotation of *GhPHD* genes in *G. hirsutum*.

| **Gene name** | **Annotation** | **Chr** | **Start** | **End** | **Strand** | **Length (bp)** |
| --- | --- | --- | --- | --- | --- | --- |
| GhPHD1 | SHL | A01 | 352464 | 356927 | - | 4464 |
| GhPHD2 | IDM1 | A01 | 101896079 | 101904325 | - | 8247 |
| GhPHD3 | IDM1 | A01 | 101920864 | 101929272 | + | 8409 |
| GhPHD4 | IDM1 | A01 | 102767570 | 102774775 | - | 7206 |
| GhPHD5 | IDM1 | A02 | 4008840 | 4016299 | + | 7460 |
| GhPHD6 | MMD1 | A02 | 57658388 | 57661121 | - | 2734 |
| GhPHD7 | MMD1 | A02 | 57834039 | 57836773 | - | 2735 |
| GhPHD8 | ATXR6 | A03 | 9484748 | 9487502 | + | 2755 |
| GhPHD9 | AL6 | A03 | 33663924 | 33671233 | + | 7310 |
| GhPHD10 | HAT3.1 | A03 | 105741279 | 105745996 | + | 4718 |
| GhPHD11 | SHL | A03 | 107094170 | 107096922 | + | 2753 |
| GhPHD12 | jade1 | A04 | 11500346 | 11501866 | + | 1521 |
| GhPHD13 | Os05g0163100 | A04 | 71153668 | 71155625 | + | 1958 |
| GhPHD14 | HAZ1 | A04 | 79260152 | 79265932 | + | 5781 |
| GhPHD15 | PRH | A05 | 309384 | 314750 | - | 5367 |
| GhPHD16 | AL3 | A05 | 3900107 | 3902820 | + | 2714 |
| GhPHD17 | HAC12 | A05 | 11127391 | 11136383 | - | 8993 |
| GhPHD18 | ALFIN-1 | A05 | 15651561 | 15656638 | + | 5078 |
| GhPHD19 | EBS | A05 | 16927596 | 16930751 | - | 3156 |
| GhPHD20 | EHD3 | A05 | 24825304 | 24829966 | + | 4663 |
| GhPHD21 | EHD3 | A05 | 24831636 | 24838222 | + | 6587 |
| GhPHD22 | ATX5 | A05 | 100427032 | 100436174 | - | 9143 |
| GhPHD23 | AL5 | A06 | 28144840 | 28148401 | + | 3562 |
| GhPHD24 | ALFIN-1 | A06 | 71053773 | 71059835 | - | 6063 |
| GhPHD25 | EBS | A06 | 111015821 | 111018520 | - | 2700 |
| GhPHD26 | PHRF1 | A06 | 117003247 | 117011745 | - | 8499 |
| GhPHD27 | HAC1 | A06 | 118478258 | 118486090 | - | 7833 |
| GhPHD28 | AL5 | A07 | 2367138 | 2370237 | + | 3100 |
| GhPHD29 | PHRF1 | A07 | 7446787 | 7450525 | + | 3739 |
| GhPHD30 | IDM1 | A07 | 18534387 | 18541412 | + | 7026 |
| GhPHD31 | IDM1 | A07 | 18675677 | 18682652 | - | 6976 |
| GhPHD32 | PTM | A07 | 24137296 | 24148440 | + | 11145 |
| GhPHD33 | PTM | A07 | 24151897 | 24162230 | - | 10334 |
| GhPHD34 | EBS | A07 | 83411217 | 83415506 | - | 4290 |
| GhPHD35 | ACX1 | A07 | 91156997 | 91162295 | - | 5299 |
| GhPHD36 | At3g20280 | A08 | 2607190 | 2611735 | - | 4546 |
| GhPHD37 | At4g10930 | A08 | 86623564 | 86633914 | - | 10351 |
| GhPHD38 | At1g33420 | A08 | 121700612 | 121704895 | - | 4284 |
| GhPHD39 | MBD9 | A09 | 8239377 | 8253833 | + | 14457 |
| GhPHD40 | EBS | A09 | 55706949 | 55709847 | + | 2899 |
| GhPHD41 | PTM | A09 | 61565963 | 61575146 | - | 9184 |
| GhPHD42 | IDM1 | A09 | 78931424 | 78939020 | + | 7597 |
| GhPHD43 | AL5 | A10 | 5366171 | 5369663 | - | 3493 |
| GhPHD44 | baz1b | A10 | 97724694 | 97730728 | + | 6035 |
| GhPHD45 | ATXR5 | A11 | 69019877 | 69024709 | - | 4833 |
| GhPHD46 | PHRF1 | A11 | 103403290 | 103407614 | + | 4325 |
| GhPHD47 | HAT3.1 | A12 | 2866756 | 2870481 | + | 3726 |
| GhPHD48 | CHR4 | A12 | 9743332 | 9745300 | + | 1969 |
| GhPHD49 | ATX3 | A12 | 75173544 | 75189791 | - | 16248 |
| GhPHD50 | MS1 | A12 | 98364964 | 98367605 | - | 2642 |
| GhPHD51 | ATX5 | A12 | 100339009 | 100347037 | + | 8029 |
| GhPHD52 | AL1 | A13 | 1858056 | 1862712 | - | 4657 |
| GhPHD53 | SHL | A13 | 3929359 | 3932603 | - | 3245 |
| GhPHD54 | Phrf1 | A13 | 71274005 | 71278329 | + | 4325 |
| GhPHD55 | IDM1 | A13 | 90586578 | 90593707 | + | 7130 |
| GhPHD56 | AL5 | A13 | 99908787 | 99911830 | + | 3044 |
| GhPHD57 | SHL | D01 | 307532 | 311646 | - | 4115 |
| GhPHD58 | IDM1 | D01 | 62432221 | 62440355 | - | 8135 |
| GhPHD59 | IDM1 | D01 | 62448388 | 62456992 | + | 8605 |
| GhPHD60 | IDM1 | D02 | 4949819 | 4957305 | + | 7487 |
| GhPHD61 | AL6 | D02 | 28345412 | 28353305 | + | 7894 |
| GhPHD62 | SHL | D02 | 69217721 | 69220616 | + | 2896 |
| GhPHD63 | MMD1 | D03 | 14803638 | 14806370 | - | 2733 |
| GhPHD64 | ATXR6 | D03 | 42920522 | 42923153 | - | 2632 |
| GhPHD65 | ATX5 | D04 | 255707 | 265537 | + | 9831 |
| GhPHD66 | Os05g0163100 | D04 | 46826823 | 46828814 | + | 1992 |
| GhPHD67 | HAZ1 | D04 | 52998570 | 53004443 | + | 5874 |
| GhPHD68 | PRH | D05 | 297739 | 302309 | - | 4571 |
| GhPHD69 | PRH | D05 | 330616 | 335978 | - | 5363 |
| GhPHD70 | AL3 | D05 | 3480360 | 3483302 | + | 2943 |
| GhPHD71 | HAC12 | D05 | 10248320 | 10259745 | - | 11426 |
| GhPHD72 | ALFIN-1 | D05 | 14683843 | 14688903 | + | 5061 |
| GhPHD73 | EBS | D05 | 15737126 | 15740367 | - | 3242 |
| GhPHD74 | EHD3 | D05 | 22761254 | 22765267 | + | 4014 |
| GhPHD75 | EHD3 | D05 | 22767653 | 22773404 | + | 5752 |
| GhPHD76 | AL5 | D06 | 14220171 | 14222608 | - | 2438 |
| GhPHD77 | AL5 | D06 | 18122783 | 18126390 | + | 3608 |
| GhPHD78 | ALFIN-1 | D06 | 40564616 | 40568436 | - | 3821 |
| GhPHD79 | EBS | D06 | 58881682 | 58883237 | - | 1556 |
| GhPHD80 | Os05g0163100 | D06 | 61304386 | 61306927 | - | 2542 |
| GhPHD81 | HAC1 | D06 | 65111315 | 65119076 | - | 7762 |
| GhPHD82 | AL5 | D07 | 2256373 | 2259187 | + | 2815 |
| GhPHD83 | PHRF1 | D07 | 7129842 | 7133440 | + | 3599 |
| GhPHD84 | IDM1 | D07 | 14908149 | 14915429 | + | 7281 |
| GhPHD85 | IDM1 | D07 | 15070280 | 15077156 | - | 6877 |
| GhPHD86 | PTM | D07 | 19134479 | 19146195 | + | 11717 |
| GhPHD87 | PTM | D07 | 19150397 | 19160154 | - | 9758 |
| GhPHD88 | EBS | D07 | 49005953 | 49010247 | - | 4295 |
| GhPHD89 | At3g20280 | D08 | 3410042 | 3414612 | - | 4571 |
| GhPHD90 | At4g10930 | D08 | 41874596 | 41884301 | - | 9706 |
| GhPHD91 | At1g33420 | D08 | 67894427 | 67898515 | - | 4089 |
| GhPHD92 | MBD9 | D09 | 7947058 | 7960172 | + | 13115 |
| GhPHD93 | EBS | D09 | 31649368 | 31652251 | + | 2884 |
| GhPHD94 | PTM | D09 | 35799934 | 35808707 | - | 8774 |
| GhPHD95 | IDM1 | D09 | 50817914 | 50825420 | + | 7507 |
| GhPHD96 | AL3 | D10 | 5053895 | 5057417 | - | 3523 |
| GhPHD97 | At1g33420 | D10 | 18084144 | 18088628 | - | 4485 |
| GhPHD98 | baz1b | D10 | 55191748 | 55202597 | + | 10850 |
| GhPHD99 | ATXR5 | D11 | 44365020 | 44369750 | - | 4731 |
| GhPHD100 | PHRF1 | D11 | 62443578 | 62447868 | + | 4291 |
| GhPHD101 | HAT3.1 | D12 | 2568434 | 2572135 | + | 3702 |
| GhPHD102 | ATX3 | D12 | 41720299 | 41728118 | - | 7820 |
| GhPHD103 | MS1 | D12 | 59241687 | 59244319 | - | 2633 |
| GhPHD104 | ATX5 | D12 | 61333000 | 61341603 | + | 8604 |
| GhPHD105 | Phrf1 | D13 | 38460226 | 38464971 | + | 4746 |
| GhPHD106 | IDM1 | D13 | 52421904 | 52428991 | + | 7088 |
| GhPHD107 | PHRF1 | D13 | 58010832 | 58014323 | + | 3492 |
| GhPHD108 | AL5 | D13 | 60075068 | 60078102 | + | 3035 |
