# Supplementary material for: Response of phytohormone mediated plant homeodomain (PHD) family to abiotic stress in upland cotton (Gossypium hirsutum spp.)
Source: BMC Plant Biol. 2021 Jan 6;21:13. doi: 10.1186/s12870-020-02787-5 (PMC7788912; doi:10.1186/s12870-020-02787-5)
Supplement: Supplementary file 6 — Additional file 6: Table S3. Transcript-features of 108 GhPHD genes [file 12870_2020_2787_MOESM6_ESM.docx]

**Table S3.** Transcript-features of the 108 *GhPHD* genes.

| **Gene name** | **Transcript Length (bp)** | **CDS Length (bp)** | **CDS GC Content (%)** | **Exon Number** | **Mean Exon Length (bp)** | **Mean Intron Length (bp)** |
| --- | --- | --- | --- | --- | --- | --- |
| GhPHD1 | 1331 | 654 | 47.7 | 5 | 266.2 | 778.8 |
| GhPHD2 | 3794 | 3102 | 41.2 | 22 | 172.5 | 212 |
| GhPHD3 | 3788 | 3093 | 41.1 | 22 | 172.2 | 220 |
| GhPHD4 | 3185 | 2448 | 41.5 | 19 | 167.6 | 223.2 |
| GhPHD5 | 4524 | 3912 | 43.4 | 8 | 565.5 | 419.4 |
| GhPHD6 | 2103 | 2103 | 43.5 | 3 | 701 | 315.5 |
| GhPHD7 | 2103 | 2103 | 43.5 | 3 | 701 | 316 |
| GhPHD8 | 1951 | 1038 | 44.3 | 6 | 325.2 | 160.8 |
| GhPHD9 | 1336 | 756 | 46 | 5 | 267.2 | 1493.5 |
| GhPHD10 | 2361 | 2361 | 41.3 | 14 | 168.6 | 181.3 |
| GhPHD11 | 1009 | 651 | 49 | 5 | 201.8 | 436 |
| GhPHD12 | 1431 | 1128 | 39.5 | 2 | 715.5 | 90 |
| GhPHD13 | 1104 | 714 | 44.5 | 5 | 220.8 | 213.5 |
| GhPHD14 | 3869 | 2880 | 41.3 | 11 | 351.7 | 191.2 |
| GhPHD15 | 2848 | 2202 | 42.6 | 11 | 258.9 | 251.9 |
| GhPHD16 | 1171 | 759 | 44.5 | 5 | 234.2 | 385.8 |
| GhPHD17 | 5560 | 5043 | 42.5 | 16 | 347.5 | 228.9 |
| GhPHD18 | 1375 | 759 | 45.3 | 5 | 275 | 925.8 |
| GhPHD19 | 1272 | 717 | 46.2 | 5 | 254.4 | 469 |
| GhPHD20 | 2204 | 1482 | 40 | 5 | 440.8 | 614.8 |
| GhPHD21 | 2795 | 1803 | 40.3 | 7 | 399.3 | 632 |
| GhPHD22 | 3550 | 3255 | 43.6 | 23 | 154.3 | 254.2 |
| GhPHD23 | 1897 | 762 | 45.8 | 5 | 379.4 | 416.3 |
| GhPHD24 | 1136 | 780 | 46.3 | 6 | 189.3 | 985.4 |
| GhPHD25 | 1399 | 675 | 44 | 5 | 279.8 | 325.3 |
| GhPHD26 | 3286 | 2613 | 42.4 | 7 | 469.4 | 679.3 |
| GhPHD27 | 4723 | 4077 | 42 | 12 | 393.6 | 282.7 |
| GhPHD28 | 1245 | 480 | 41.9 | 5 | 249 | 463.8 |
| GhPHD29 | 2409 | 2202 | 42 | 3 | 803 | 661 |
| GhPHD30 | 3976 | 3744 | 40.7 | 12 | 331.3 | 277.3 |
| GhPHD31 | 3600 | 2850 | 44.1 | 18 | 200 | 198.6 |
| GhPHD32 | 5899 | 4857 | 40.9 | 10 | 589.9 | 582.9 |
| GhPHD33 | 5925 | 4857 | 41 | 10 | 592.5 | 489.9 |
| GhPHD34 | 1304 | 651 | 47.3 | 5 | 260.8 | 746.5 |
| GhPHD35 | 2194 | 966 | 41.7 | 9 | 243.8 | 388.1 |
| GhPHD36 | 2790 | 2469 | 44.3 | 7 | 398.6 | 292.7 |
| GhPHD37 | 4674 | 3918 | 41.2 | 15 | 311.6 | 405.5 |
| GhPHD38 | 2374 | 2118 | 44 | 5 | 474.8 | 477.5 |
| GhPHD39 | 8029 | 6696 | 42.3 | 10 | 802.9 | 714.2 |
| GhPHD40 | 1138 | 681 | 44.8 | 5 | 227.6 | 440.3 |
| GhPHD41 | 5993 | 5058 | 42 | 10 | 599.3 | 354.6 |
| GhPHD42 | 4590 | 3720 | 42.7 | 9 | 510 | 375.9 |
| GhPHD43 | 1157 | 762 | 45 | 5 | 231.4 | 584 |
| GhPHD44 | 2194 | 1596 | 42.3 | 6 | 365.7 | 768.2 |
| GhPHD45 | 1651 | 1170 | 44.9 | 7 | 235.9 | 530.3 |
| GhPHD46 | 3663 | 2412 | 43.5 | 4 | 915.8 | 220.7 |
| GhPHD47 | 2868 | 2556 | 39.6 | 8 | 358.5 | 122.6 |
| GhPHD48 | 1295 | 639 | 40.7 | 3 | 431.7 | 337 |
| GhPHD49 | 4118 | 3060 | 41.6 | 26 | 158.4 | 485.2 |
| GhPHD50 | 2114 | 1968 | 41.3 | 3 | 704.7 | 264 |
| GhPHD51 | 3603 | 3276 | 43.3 | 23 | 156.7 | 201.2 |
| GhPHD52 | 1365 | 714 | 45.5 | 5 | 273 | 823 |
| GhPHD53 | 1139 | 651 | 47.8 | 5 | 227.8 | 526.5 |
| GhPHD54 | 2846 | 2151 | 41.7 | 3 | 948.7 | 169 |
| GhPHD55 | 4301 | 4104 | 41 | 7 | 614.4 | 471.5 |
| GhPHD56 | 1279 | 765 | 47.1 | 5 | 255.8 | 441.3 |
| GhPHD57 | 1082 | 654 | 47.7 | 5 | 216.4 | 758.3 |
| GhPHD58 | 3686 | 3096 | 41.2 | 22 | 167.5 | 211.9 |
| GhPHD59 | 3847 | 3096 | 41.4 | 23 | 167.3 | 216.3 |
| GhPHD60 | 4553 | 3900 | 43.3 | 8 | 569.1 | 419.1 |
| GhPHD61 | 1327 | 873 | 45 | 4 | 331.8 | 2151.3 |
| GhPHD62 | 1150 | 651 | 48.7 | 5 | 230 | 436.5 |
| GhPHD63 | 2100 | 2100 | 43.6 | 3 | 700 | 316.5 |
| GhPHD64 | 1848 | 1038 | 44.4 | 6 | 308 | 156 |
| GhPHD65 | 4186 | 3255 | 43.3 | 23 | 182 | 256.6 |
| GhPHD66 | 1159 | 714 | 44.5 | 5 | 231.8 | 208.3 |
| GhPHD67 | 3719 | 2838 | 41.1 | 10 | 371.9 | 239.4 |
| GhPHD68 | 2274 | 2055 | 43.4 | 12 | 189.5 | 208.8 |
| GhPHD69 | 2853 | 2202 | 42.8 | 11 | 259.4 | 251 |
| GhPHD70 | 1374 | 759 | 45.2 | 5 | 274.8 | 392.3 |
| GhPHD71 | 6803 | 5196 | 42.7 | 17 | 400.2 | 288.9 |
| GhPHD72 | 1356 | 759 | 45.5 | 5 | 271.2 | 926.3 |
| GhPHD73 | 1339 | 675 | 46.7 | 5 | 267.8 | 475.8 |
| GhPHD74 | 1664 | 1104 | 41.8 | 4 | 416 | 768 |
| GhPHD75 | 2446 | 1806 | 40.6 | 6 | 407.7 | 661.2 |
| GhPHD76 | 726 | 726 | 43 | 4 | 181.5 | 570.7 |
| GhPHD77 | 1925 | 762 | 46.1 | 5 | 385 | 420.8 |
| GhPHD78 | 1216 | 759 | 45.7 | 5 | 243.2 | 651.3 |
| GhPHD79 | 561 | 561 | 43.5 | 4 | 140.3 | 331.7 |
| GhPHD80 | 1177 | 714 | 46.5 | 5 | 235.4 | 341.3 |
| GhPHD81 | 4688 | 4071 | 42 | 12 | 390.7 | 279.5 |
| GhPHD82 | 1450 | 711 | 43 | 5 | 290 | 341.3 |
| GhPHD83 | 2281 | 2031 | 41.9 | 3 | 760.3 | 659 |
| GhPHD84 | 4433 | 4149 | 40.9 | 12 | 369.4 | 258.9 |
| GhPHD85 | 3489 | 2850 | 44.1 | 18 | 193.8 | 199.3 |
| GhPHD86 | 5905 | 4962 | 41.2 | 9 | 656.1 | 726.5 |
| GhPHD87 | 5249 | 4857 | 40.9 | 10 | 524.9 | 501 |
| GhPHD88 | 1282 | 651 | 46.9 | 5 | 256.4 | 753.3 |
| GhPHD89 | 2806 | 2469 | 44.2 | 7 | 400.9 | 294.2 |
| GhPHD90 | 4458 | 3906 | 41.6 | 14 | 318.4 | 403.7 |
| GhPHD91 | 2371 | 2118 | 43.9 | 5 | 474.2 | 429.5 |
| GhPHD92 | 6549 | 6549 | 42.5 | 12 | 545.8 | 596.9 |
| GhPHD93 | 1118 | 681 | 44.9 | 5 | 223.6 | 441.5 |
| GhPHD94 | 5733 | 5058 | 42.1 | 9 | 637 | 380.1 |
| GhPHD95 | 4507 | 3714 | 42.6 | 9 | 500.8 | 375 |
| GhPHD96 | 1185 | 762 | 44.9 | 5 | 237 | 584.5 |
| GhPHD97 | 2646 | 2091 | 43.6 | 5 | 529.2 | 459.8 |
| GhPHD98 | 2159 | 1512 | 42.7 | 6 | 359.8 | 1738.2 |
| GhPHD99 | 1541 | 1158 | 45.3 | 7 | 220.1 | 531.7 |
| GhPHD100 | 3275 | 2439 | 43.3 | 5 | 655 | 217.3 |
| GhPHD101 | 2867 | 2580 | 39.7 | 8 | 358.4 | 119.3 |
| GhPHD102 | 3615 | 3060 | 41.7 | 23 | 157.2 | 191.1 |
| GhPHD103 | 2107 | 1968 | 41.4 | 3 | 702.3 | 263 |
| GhPHD104 | 3742 | 3276 | 43.2 | 24 | 155.9 | 211.4 |
| GhPHD105 | 3275 | 2670 | 41.6 | 4 | 818.8 | 330 |
| GhPHD106 | 4337 | 3918 | 40.8 | 7 | 619.6 | 458.5 |
| GhPHD107 | 2883 | 2406 | 42.3 | 3 | 961 | 131 |
| GhPHD108 | 1395 | 759 | 46.8 | 5 | 279 | 410 |
