# Supplementary material for: Response of phytohormone mediated plant homeodomain (PHD) family to abiotic stress in upland cotton (Gossypium hirsutum spp.)
Source: BMC Plant Biol. 2021 Jan 6;21:13. doi: 10.1186/s12870-020-02787-5 (PMC7788912; doi:10.1186/s12870-020-02787-5)
Supplement: Supplementary file 10 — Additional file 10: Table S7. Primers for RT-qPCR in this study [file 12870_2020_2787_MOESM10_ESM.docx]

**Table S7.** Primers for RT-qPCR in this study.

| **Gene** | **Forward primer (5'-3')** | **Reverse primer (5'-3')** |
| --- | --- | --- |
| histone3 | TCAAGACTGATTTGCGTTTCCA | GCGCAAAGGTTGGTGTCTTC |
| GhPHD5 | GAGTGATACGACGAGTATGGGTGT | GACTCAGAACCGGCAAGTGTGT |
| GhPHD18 | TTGTGGGGCTTGTGGAGATAGC | TTGCCATGGAACCATCTCTCGC |
| GhPHD23 | GGACTGGTTGTCTTTGGTTGC | TGTTTCTTAGTTGCCCCTGT |
| GhPHD24 | TTGCTTGCTGTTGCTTTCTA | CTTTTGCTGCTGCCATTGTG |
| GhPHD34 | TTGTTCGTCCTGGTGATTGCGT | ATGGTCGGCCTCGATCTTCTCT |
| GhPHD40 | CCTTAGCTCCCAAGTCCAAA | CCTTCCTCCAAGTGATTCCT |
| GhPHD43 | CCAACTCAAAAGCGCGGGAATC | GCTCGTCTTCGTCTTCCTCGTC |
| GhPHD72 | CCAAATGAGACATGGGAAGT | CCAAAATAGAAAGCAACAGC |
| GhPHD77 | TGGAGAGACGTTATGTGGGGCT | CGCCCTTGCCGGTGTTATCTTA |
| GhPHD80 | GAGGATGAAGAAGAGCAGGGCG | TCATTTCTGCCTTCGCTGGTGT |
| GhPHD88 | CGTCCTGGTGATTGTGTGCTGA | ATGGTCGGCCTCGATCTTCTCT |
| GhPHD107 | TGCTCAACTCTATCAGTGCCGC | GGTACCATGTGAACCTCGCTCC |
